# RTHistory: An automated tool for the extraction and presentation of radiotherapy course history for continuity of care applications

**DOI:** 10.1002/acm2.70910

**Published:** 2026-11-14

**Authors:** Justin B. Hink, Yasin Abdulkadir, Andrew Krupien, Peter Boyle, Daniel A. Low, Jack Neylon, James Lamb

**Affiliations:** ^1^ Department of Radiation Oncology, David Geffen School of Medicine at UCLA University of California, Los Angeles Los Angeles California USA

**Keywords:** automation, DICOM‐RT, re‐irradiation

## Abstract

**Background:**

Transfer of radiation treatment records is a common task for radiotherapy clinics, as treatment history impacts future treatment planning, including prescription dose, fractionation, and normal tissue constraint selections. Currently, gathering radiation records relies on manual searches within treatment planning databases and Record and Verify (R&V) systems, a process that is time‐consuming and subject to human error. Particularly challenging is discerning which plans were treated, and for how many fractions, in cases of treatment adaptation or early termination.

**Purpose:**

In this technical note, we present *RTHistory*, an open‐source software tool that automates the extraction process for R&V systems that implement DICOM Query/Retrieve and summarizes the treatment history in a format optimized for efficient clinical readability and visual triage.

**Methods:**

RTHistory was configured to query multiple clinical systems through the DICOM protocol. The query process begins with downloading and analyzing the RTRecords, which document beam delivery. From these, internal DICOM object references are followed to gather the relevant planning files, including dose, simulation scan, and structure set. RTHistory then parses these DICOM files to create a treatment summary PDF report that describes the plan labels, prescription doses, treatment start and end dates, number of fractions treated, and a graphical representation of the dose washes of the treated plans. Rigorous software unit testing and error handling procedures were implemented to ensure reliability and accuracy of the extracted data.

**Results:**

RTHistory was evaluated on a clinical cohort of 61 patients comprising 177 distinct treatment plans across six anatomical sites from our institutional database, which included the ARIA/Eclipse R&V system and treatment planning system. Manual verification against clinical ARIA and Eclipse records confirmed 100% agreement in reconstructing delivered DICOM‐RT objects (177/177 plans), with none of 439 undelivered plans included (specificity: 100%; 95% CI: 99.2%–100%). Performance testing for patients with at least one treatment planned in Eclipse (*n* = 51 patients) yielded a median overall patient run time of 115.4 s (aggregate per‐plan: 59.5 s, 95% bootstrap confidence interval: 45.6–72.1 s), which scaled directly with the number of CT series (93.4 ± 20.0 s per series). In a direct comparison across 42 patients, manual abstraction required a mean active hands‐on time of 342.0 ± 378.0 s (median: 192.0 s, range: 90.0–2214.0 s), whereas the software ran unattended with a mean execution time of 228.0 ± 282.0 s (median: 114.0 s, range: 48.0–1318.6 s).

**Conclusion:**

RTHistory offers a solution to improve continuity of care by facilitating accurate information sharing through the automated extraction and presentation of treatment history. Looking ahead, integration into institutional workflows holds promise for enhancing patient care and decision‐making in oncological treatment, while reducing the manual burden on clinical staff and mitigating transcription risks in re‐irradiation workflows.

## 1 INTRODUCTION

Radiation therapy relies on a complex set of workflows and data transfers to facilitate personalized treatment planning. As cancer survival rates improve, an increasing number of patients are presenting for a second course of radiation therapy^1^, whether a retreatment or after the development of a second primary tumor. For these patients, the treatment planning process must account for the previously delivered dose^2^, ^3^. Radiation oncologists and medical physicists collaborate to determine a deliverable dose distribution that adequately treats the new target volume while minimizing cumulative toxicity to surrounding healthy tissue^1^. Overly conservative organ at risk (OAR) dose estimates unnecessarily risk compromising target coverage and potential treatment failure; conversely, disregarding the prior dose entirely risks increased normal tissue toxicity probabilities^4^. Finding a precise and accurate balance is therefore crucial for patient safety and the overall probability of treatment success, especially given the considerable clinical effort and cognitive workload inherent to re‐irradiation review^5^.

To ensure that a clinic can generate a safe and effective treatment plan for a patient requiring retreatment, standard of care requires procuring a summary of previous treatments from the original treating institution. However, the format and detail of these summaries are almost entirely dependent on the individual clinics’ reporting standards. Currently, the abstraction of treatment history from available electronic records relies largely on manual searches within treatment planning databases and Record and Verify (R&V) systems. This unstandardized, manual procedure is time‐consuming and potentially susceptible to human error. Possible errors include omitted treatments, missing prescription doses, and the accidental inclusion of planned but ultimately undelivered treatments. Discerning exactly which plans were treated, and for how many fractions, is particularly challenging in cases of early termination or mid‐course treatment adaptation.

While commercial Oncology Information Systems (OIS) such as Elekta MOSAIQ (Elekta AB, Stockholm, Sweden), RaySearch RayCare (RaySearch Laboratories AB, Stockholm, Sweden), and Varian ARIA (Varian Medical Systems, Inc., Palo Alto, CA) incorporate summary dashboards, these tools require direct access to the proprietary database schemas, preventing automated, standardized aggregation across multi‐vendor networks or transferring institutions. Furthermore, enterprise informatics initiatives^6^ are architected for large‐scale retrospective population registries and national radiation safety tracking rather than on‐demand, point‐of‐care clinical review. Early DICOM‐RT electronic patient record implementations^7^, ^8^ demonstrated the feasibility of extracting per‐fraction treatment timelines but relied on complex institutional web infrastructure.

To address these challenges, we developed an automated software tool, *RTHistory*, designed to query, retrieve, and summarize a patient's complete radiotherapy treatment history. The software relies on the Digital Imaging and Communications in Medicine (DICOM) standard^9^, a widely adopted, non‐proprietary data interchange protocol for biomedical imaging and metadata. Because it uses standard DICOM services rather than proprietary database schemas, the approach should be applicable to any R&V system that implements DICOM Query/Retrieve, although the present implementation has been tested only within an ARIA/Eclipse environment. By systematically traversing the DICOM‐RT^8^ relational tree^10^ (Figure 1), our tool connects the various disparate files that comprise a complete patient record. The purpose of this technical note is to describe the development, network architecture, performance testing, and clinical implementation of this automated treatment history extraction software.

**FIGURE 1:**
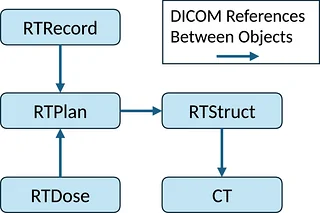
The DICOM‐RT relational tree for radiotherapy objects. To assemble a complete patient treatment record (i.e., the DICOM objects representing a plan and its delivery), an automated retrieval system must traverse DICOM‐standard internal references (shown as arrows). The RTRecord anchors the delivered treatment by pointing directly to the RTPlan. The RTDose object also points to the RTPlan it was calculated for. In turn, the RTPlan points to the RTStruct used for planning, and the RTStruct ultimately points to the CT image series.

## 2 METHODS

### 2.1 Software architecture and clinical deployment

RTHistory was developed in Python (Version 3.13.13) for the Windows environment and utilizes the pynetdicom package. RTHistory is distributed as an open‐source package under the permissive MIT license hosted on GitLab at https://gitlab.com/justin‐hink/RTHistory and on Zenodo at https://doi.org/10.5281/zenodo.23960495. The codebase is organized into decoupled, modular subsystems (main.py, DataIngestion_cli.py, config.py, logger_setup.py, QueryRetrieveSCU_rosamllib.py, StoreSCP_rosamllib.py, TaskManager_rosamllib.py, and PdfParser_rosamllib.py). Software dependencies (pydicom, SimpleITK, numpy, matplotlib, reportlab, sqlalchemy, pandas, tabulate, and rosamllib) are version‐pinned in a requirements.txt file to ensure reproducible deployment. This design choice enables straightforward deployment across clinical workstations by allowing users to install the software and its dependencies into a local Python environment using the standard *pip* package manager.

After initializing RTHistory, the user is prompted to populate information for local configuration, saving it to a local file. These configuration parameters include the Application Entity (AE) Titles, IP addresses, and ports for the clinical R&V server and the application itself. Establishing the local Service Class Provider (SCP) requires the host workstation to bind a designated listening port and open it on the local firewall. Depending on institutional IT policies, this network requirement may necessitate local administrator privileges or institutional IT approval prior to deployment.

### 2.2 Security infrastructure and IT compliance

To comply with hospital cybersecurity standards, DICOM communication is restricted exclusively to internal clinical local area networks (LAN) or secure VLANs behind institutional firewalls. The clinical Picture Archiving and Communication System (PACS) and R&V servers enforce network security by requiring administrative whitelisting of the client AE Title, IP address, and listening port before accepting associations, preventing unauthorized network endpoints from querying clinical archives. Furthermore, RTHistory operates under a strict read‐only, least‐privilege model (C‐FIND and C‐MOVE DICOM Message Service Element [DIMSE] services), possessing no database write, update, or delete permissions. All DICOM parsing, image processing, and PDF rendering occur strictly in local workstation storage without transmitting Protected Health Information (PHI) to external or cloud services.

### 2.3 DICOM query, retrieve, and task management

Networking is handled via standard DICOM DIMSE services including C‐FIND, C‐MOVE, and C‐STORE. Because DICOM Query/Retrieve hierarchy implementations can vary widely between vendors, this software's specific C‐FIND logic was tailored to and validated exclusively within an ARIA/Eclipse ecosystem version 18.00.10^11^. Attempting to query alternative proprietary databases may require modifications to the query structure due to differing internal database indexing.

Unlike manual searches that often begin with the treatment plan, our automated retrieval process begins by systematically searching the R&V system for the patient's Radiotherapy Treatment Records (i.e., the RTRecord DICOM object). This initial step ensures that the software only queries data for treatment plans corresponding to the fractions that were delivered. To prevent the retrieval of patient‐specific quality assurance (QA) deliveries executed under clinical treatment mode, RTHistory verifies the ApprovalStatus tag to filter out plans designated as verification RTPlans.

From these RTRecords, the software extracts the necessary reference tags to collect the associated RTPlan, RTStruct, RTDose, and computed tomography (CT) simulation images. Data retrieval follows strict hierarchical logic to ensure only treatment data related to delivered treatment fractions are pulled. The Service Class User first initiates a C‐FIND request targeting the R&V server for all RTRecords (SOP Class UID 1.2.840.10008.5.1.4.1.1.481.4) associated with the input medical record number. Using the ReferencedSOPInstanceUID from the returned records, the software identifies the specific RTPlans that were treated and transfers them to the SCP via a C‐MOVE request. To transfer the associated RTDose objects, RTHistory utilizes the identified RTPlan's SOPInstanceUID as the reference tag in the query dataset. Subsequently, the target RTPlan is parsed to extract the ReferencedStructureSetSequence to identify and move the associated RTStruct to the SCP. Finally, the RTStruct's ContourImageSequence is parsed to extract the ReferencedSOPInstanceUID from the DICOM header of one slice of the parent volumetric imaging series. This imaging series is then moved to the SCP using the SeriesInstanceUID of the slice found.

To handle interrupted fraction deliveries where partial beams are delivered across multiple sessions, the software parses the CurrentFractionNumber across all RTRecord objects into a unique set, ensuring that each delivered fraction is counted exactly once in the cumulative total. To mitigate transient network packet drops during large volumetric image transfers, the task manager incorporates an automated retry routine that attempts up to 10 reconnections per file before raising a terminal error.

As objects are retrieved sequentially, the local Service Class Provider dynamically saves and organizes the incoming DICOM files into an isolated temporary directory structured by StudyInstanceUID, Modality, and SeriesInstanceUID. Figure 2 illustrates the strict hierarchical data retrieval workflow used by RTHistory, detailing the specific DICOM reference tags utilized at each step to navigate the relational tree and extract the necessary clinical parameters.

**FIGURE 2:**
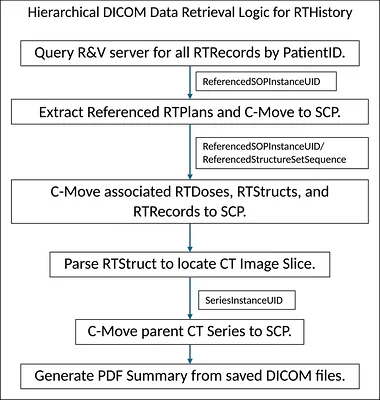
RTHistory's hierarchical DICOM data retrieval logic. The automated extraction workflow begins with the retrieval of Radiotherapy Treatment Records (RTRecords) to ensure only delivered plans are processed. Grey boxes denote the specific DICOM reference tags parsed to identify and collect the associated files (RTPlan, RTDose, RTStruct, and CT imaging). Following successful data retrieval, the DICOM dataset is processed to generate the treatment summary PDF report.

### 2.4 Data extraction and report generation

Upon completion of the network transfers, the software parses the retrieved local DICOM files to compile the patient's comprehensive clinical history. This extracted data is synthesized into a standardized, human‐readable Treatment History Report in PDF format, using the ReportLab library^12^. This document features a chronological timeline of treatments, detailed plan parameters, and a spatial representation of the historical dose distribution. Finally, the Treatment History Report and all associated DICOM files are compressed into a single archive to create the final radiotherapy history package. An example Treatment History Report excerpt is shown in Figure 3, and a full version is included as Figure S1.

**FIGURE 3:**
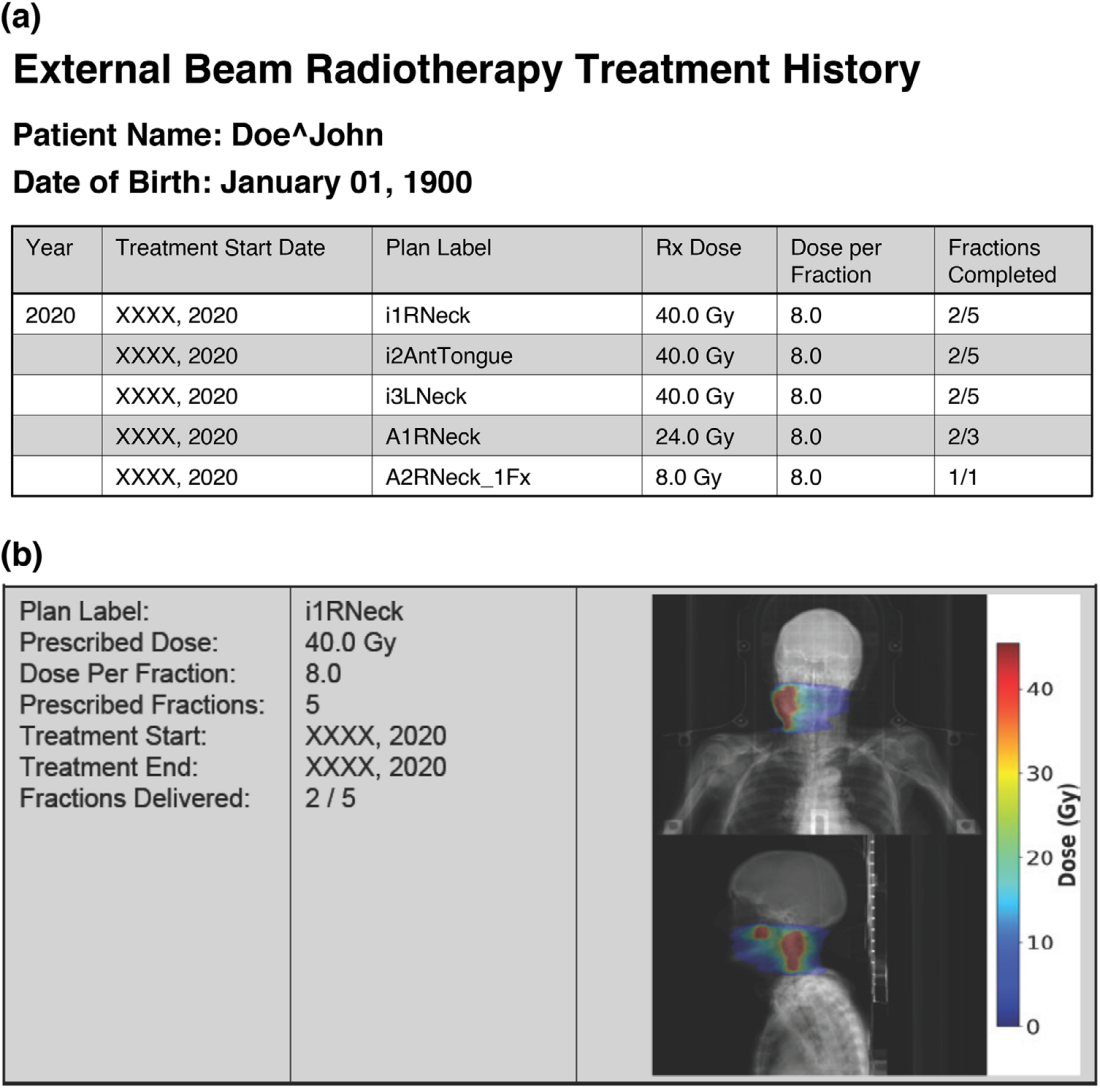
Example output (excerpted) of the Treatment History Report. (a) A chronological timeline detailing the treatment start dates, plan labels, prescribed doses, and the exact number of fractions completed for a highly complex, adapted treatment course. (b) Plan‐specific tabular summaries accompanied by 2D projections of the computed tomography (CT) simulation images. The corresponding maximum intensity dose wash (jet colormap, thresholded at 10% maximum dose) is overlaid on the CT alongside an absolute dose colorbar in gray (Gy), providing immediate spatial and anatomical context.

To accurately build the patient's chronological timeline and capture the clinical intent, RTHistory maps both fractionation and prescription data. First, the application iterates through the TreatmentSessionBeamSequence of the RTRecords. The CurrentFractionNumber reveals if fractions were omitted and establishes the precise treatment start and end dates. Planned fraction counts are extracted directly from the NumberOfFractionsPlanned attribute located within the FractionGroupSequence of the referenced RTPlan, allowing the report to present delivered versus planned fraction ratios (e.g., 2/5). The report identifies treatments by their RTPlanLabel tag within the RTPlan. Next, target and OAR prescriptions are extracted from the RTPlan's DoseReferenceSequence. To distinguish between the intended therapeutic dose and maximum dose limits, the application differentiates between the TargetPrescriptionDose and the OrganAtRiskMaximumDose. Plans utilizing the OAR‐type prescriptions are automatically flagged with an asterisk in the final report to alert the reviewing physicist.

To provide spatial context for the Treatment History Report, a visual representation of the dose distribution is automatically generated. The planning CT image and the associated RTDose grid are imported via SimpleITK^13^ and resampled to an isotropic spacing. Coronal and sagittal 2D representations of the patient anatomy are generated using a mean intensity projection of the CT. The 3D dose grid is projected along the corresponding orthogonal axes using a maximum intensity projection (MIP). These 2D dose projections are converted to a colormap, scaled from 10% to 100% of the maximum planned dose, and overlaid onto the grayscale CT projections using Matplotlib^14^. An absolute dose colorbar in units of gray (Gy) is rendered adjacent to the orthogonal views for plans with an associated dose grid to allow immediate visual calibration.

### 2.5 Clinical safety, auditing, and output packaging

Robust background logging is implemented via SQLAlchemy^15^ and a local SQLite database to audit the high‐risk nature of radiotherapy data transfers. Every C‐FIND, C‐MOVE, and parsing operation is time‐stamped and recorded.

To prioritize data integrity over extraction yield, the application employs a strict fail‐closed architecture, meaning it will halt execution rather than risk presenting an incomplete treatment history. In practice, RTHistory differentiates between expected clinical edge cases (where partial reporting is handled safely) and unexpected data corruption (which requires a hard abort).

Specific error‐handling routines are implemented to identify non‐standard or legacy machine configurations that lack standard volumetric image‐to‐dose spatial mapping. By parsing the TreatmentMachineName, Manufacturer, and RadiationType tags, RTHistory dynamically detects these cases, including treatments delivered on systems connected to an external R&V system (e.g., ViewRay MRIdian [ViewRay Inc., Denver, CO], TomoTherapy [Accuray Inc., Madison, WI], or Varian Ethos). In clinical practice, treatments delivered using these systems are often tracked in the primary R&V system (e.g., ARIA) to maintain centralized records, which creates RTRecords linked to “dummy” RTPlans in the primary R&V. These dummy plans lack the dosimetric information of an actual plan. Thus, the application skips the automated image overlay for that specific plan and prints a note that a manual export would be required to provide full dosimetric information for that plan. Primary planning objects for these independent platforms are maintained within external proprietary databases and are considered out of scope for automated DICOM retrieval.

Conversely, if the application encounters a broken DICOM reference chain, it immediately halts processing for that patient. A broken DICOM reference chain typically presents as a missing RTDose or RTStruct file for a standard delivered plan. It is important to note that this is a rare occurrence caused by database‐level data corruption or a completely missing file within the primary R&V or Treatment Planning System (TPS) itself, rather than a failure of the extraction algorithm to retrieve an existing file. Because such a missing file implies the automated extraction can no longer be trusted to provide a comprehensive history, RTHistory intentionally aborts execution. In such a case, the specific point of failure is logged and a prominent warning alerts the user that the automated run has failed, and that a full manual extraction is required. This binary trust model ensures that a partial or unverified treatment record is never inadvertently presented as a complete history.

Following a successful extraction and generation of the Treatment History Report, the application packages the data. An output folder is created in the current working directory where the application was started. The application then creates a compressed archive named according to the patient's medical record number to encapsulate both the DICOM files and the Treatment History Report. Finally, the temporary working directories are purged to ensure data privacy and maintain storage hygiene on the host workstation.

### 2.6 Software validation and performance evaluation

To evaluate the quantitative accuracy and retrieval performance of RTHistory, a retrospective validation cohort of 61 patients comprising 177 distinct treatment plans was retrieved from our institutional database. Within our clinical network environment, clinical physics assistants (CPAs) executed the software across these historical cases and performed independent manual verification. For every case, generated PDF reports and underlying DICOM objects were cross‐referenced directly against ARIA and Eclipse records to audit plan completeness, fraction concordance, and retrieval specificity. Additionally, active hands‐on manual chart abstraction times were recorded across 42 patients to establish a direct comparative baseline against automated execution. Manual abstraction times were recorded by stopwatch, whereas automated execution times were recorded by the software's internal timing; the time required to launch the software and review the generated report was not formally measured. Confidence intervals for proportions were calculated using the exact binomial method.

## 3 RESULTS

The clinical evaluation cohort comprised 61 patients and 177 distinct treatment plans across six anatomical sites, encompassing a broad range of delivery techniques, re‐irradiations, adaptive replans, and multi‐vendor delivery platforms (Table 1).

**TABLE 1:** Clinical validation cohort characteristics (*n* = 61 patients, 177 treatment plans).

Category	Characteristic/Subtype	Plan count (*n* = 177)
Treatment technique	3D conformal radiotherapy (3DCRT)	34
	Intensity‐modulated radiotherapy (IMRT)	2
	Volumetric modulated arc therapy (VMAT)	80
	Stereotactic radiosurgery (SRS)	17
	Stereotactic body radiotherapy (SBRT)	43
	Electron therapy	1
Anatomical site	Thorax	67
	Head and neck	27
	Pelvis	25
	Cranial	23
	Abdomen	18
	Extremity	17
Clinical complexity	Re‐irradiation plans (> 30 days apart in same region)	35
	Adaptive plans	10
	Plans with fraction interruption	1
	ViewRay plans (Independent R&V)	12
	TomoTherapy plans (Independent R&V)	14
	Ethos plans (Independent R&V)	16
	Legacy linac treatment (Decommissioned Machine)	1

Across the 61 patients, the clinical databases contained 616 plans, of which 177 were delivered and 439 were undelivered. Undelivered plans comprised patient‐specific QA plans and unused trial versions of treatment plans. Manual verification against clinical ARIA and Eclipse records demonstrated 100% agreement in reconstructing delivered DICOM‐RT objects (177/177 plans; 95% CI: 97.9%–100%), and none of the 439 undelivered plans were included in any report (specificity: 100%; 95% CI: 99.2%–100%). All delivered fraction counts, plan labels, and prescription attributes showed 100% concordance with clinical records. When encountering plans treated on platforms with independent R&V systems (ViewRay, TomoTherapy, Ethos; *n* = 42 plans), the software correctly bypassed dose overlay generation and populated a manual export alert without execution failure.

Execution timing was analyzed across all patient treatment history extractions with at least one Eclipse plan (*n* = 51), yielding a median overall patient runtime of 115.4 s (interquartile range [IQR]: 86.2–225.0 s; mean: 211.3 ± 228.3 s). On an aggregate per‐plan basis, the processing rate was 59.5 s per plan (95% bootstrap confidence interval over 10,000 resamples: 45.6–72.1 s). Timing breakdown revealed that image transfer is the primary operational bottleneck, with CT series C‐MOVE transfers accounting for 55.4% of total pipeline execution time (mean per‐patient CT share: 53.5 ± 12.6%; isolated CT transfer rate: 49.3 ± 13.6 s per series, median: 51.7 s). Normalizing overall patient runtime by the number of simulation CT series yielded a predictive metric of 93.4 ± 20.0 s per CT series (median: 91.7 s, range: 43.5–141.9 s), demonstrating that total runtime scales directly with the number of unique CT series rather than fraction or plan counts.

In a direct workflow comparison across 42 patients, manual chart abstraction by clinical physics assistants required a mean active hands‐on time of 342.0 ± 378.0 s (median: 192.0 s, range: 90.0–2214.0 s). Complex multi‐course and adaptive cases routinely demanded 600.0 to 1380.0 s of continuous manual effort. For the same patients, RTHistory required a mean execution time of 228.0 ± 282.0 s (median: 114.0 s, range: 48.0–1318.6 s). These measures are not directly equivalent: manual time represents active staff effort, whereas automated execution proceeds unattended once launched, with active input limited to starting the software and reviewing the generated report. Therefore, the principal benefits are the reduction in active hands‐on time and systematic query and retrieval of plans, rather than a reduction in elapsed time.

To illustrate the clinical utility of this verification process, Figure 3 demonstrates the software's output for a highly complex patient case involving mid‐course adaptations. In this scenario, the patient had three initial plans that were terminated early, followed by two subsequent adaptive plans. Manually extracting this specific history from the R&V system is prone to transcription errors. A manual review might easily lead to the dangerous assumption that the patient received the full prescribed dose for all five planned treatments. Because our software prioritizes the retrieval of RTRecords first, the generated timeline accurately reflects the early terminations, capturing the exact fraction count for both the aborted and adaptive treatment plans (Figure 3a). This ensures the receiving clinician is presented with the true, cumulative dose the patient received, complete with immediate spatial context (Figure 3b), rather than a theoretical planned maximum. A complete generated treatment summary PDF report is provided in the Figure S1.

## 4 DISCUSSION

A primary clinical value of this software lies in its ability to reliably distinguish between planned and delivered treatments. By initiating the hierarchical search query with the RTRecord rather than the RTPlan, the software guarantees that early terminations and mid‐course adaptations are accurately reflected in the final summary. This mechanism prevents the dangerous clinical scenario of a receiving institution modifying a new prescription based on a historical plan that was not fully delivered. By replacing variable manual chart abstraction with a deterministic standard, RTHistory also reduces active clinical workload in re‐irradiation workflows and is expected to reduce inter‐observer variability.

Furthermore, automating the spatial dose overlay provides the receiving physician with immediate anatomical context. While the Treatment History Report PDF lacks detailed dosimetric descriptors, such as Dose‐Volume Histograms (DVHs) and precise isodose lines, required for evaluating complex overlapping treatments, the document serves as a highly efficient first‐pass triage tool. Treatment planners can quickly review the treated anatomic regions to determine if previous radiation fields intersect with the current target volume, and only if so, proceed with more detailed analysis of DICOM data using third‐party software for deformable registration and composite dose summation.

Because RTHistory relies only on standard DICOM DIMSE services and DICOM‐RT reference tags, it should function with any R&V system that correctly implements DICOM Query/Retrieve. However, it has only been tested within an ARIA/Eclipse environment, and because Query/Retrieve implementations differ between vendors, adjustment to the software may be required before use with other R&V systems. The application requires DICOM‐RT‐compliant reference links. In older archival cases, missing DICOM reference UIDs or non‐standard private tags can disrupt reference links, causing the software to immediately abort execution and alert the user.

Additionally, legacy database serialization latency can impact runtime: while modern plans query within seconds, queries for legacy treatments from decommissioned linacs (e.g., dating to 2008) required up to roughly 22.0 minutes (1318.6 s) as the vendor R&V dynamically reconstructed historical records for export.

Clinical scope is also constrained when encountering non‐standard platforms or alternative modalities: dedicated treatment delivery systems operating independent R&Vs (ViewRay, TomoTherapy, Ethos) store primary planning objects in external databases requiring manual export for full volumetric dosimetry. Additionally, the software is currently limited to external beam treatments and does not support the distinct DICOM data structures associated with brachytherapy. However, these limitations do not result in silent failures; the system maintains a high standard of clinical safety by intentionally halting the automated process or flagging “dummy” plans and alerting the user whenever an unsupported chain is detected. Furthermore, no formal usability or inter‐user consistency assessment was performed, and the time required to launch the software and review its output was not formally measured. Finally, while technical accuracy was verified with 100% agreement, a prospective study evaluating downstream impacts on physician decision‐making and planning efficiency represents an important next step. Future work will focus on expanding compatibility with other major OIS and developing custom parsing logic to incorporate non‐standard delivery platforms.

## 5 CONCLUSION

The automated extraction tool presented in this study addresses a critical vulnerability in the continuity of care for radiotherapy patients: manual data extraction and summary. As the need for retreatments and complex adaptations increases, the reliance on manual processes from treatment planning and R&V systems introduces unacceptable risks of human transcription error. By shifting the burden of data collection to an automated DICOM‐based workflow, clinics can ensure that transferring patients are accompanied by an accurate and comprehensive treatment history.

## AUTHOR CONTRIBUTIONS

Justin B. Hink conceptualized the study, served as the lead developer for the RTHistory tool, managed its integration into the clinical workflow alongside Andrew Krupien and James Lamb, and wrote the primary manuscript draft. Yasin Abdulkadir, Peter Boyle, and Jack Neylon contributed to the software development and codebase refinement. Daniel A. Low, James Lamb, and Jack Neylon provided overall project supervision and clinical guidance. All authors provided critical review, edited the manuscript, and approved the final version for submission.

## CONFLICT OF INTEREST STATEMENT

The authors declare no conflicts of interest.

## ETHICS STATEMENT

This work was conducted as a quality improvement initiative evaluating a software tool supporting routine clinical workflows, using data generated during standard‐of‐care radiotherapy treatment. Under institutional policy governing quality improvement activities, this project was determined not to constitute human subjects research and did not require Institutional Review Board review.

## Supporting information



Supporting Information

## Data Availability

The RTHistory source code (v1.0.0) developed for this study is open‐sourced under the MIT license and publicly available on Zenodo at https://doi.org/10.5281/zenodo.23960495 and on GitLab at https://gitlab.com/justin‐hink/RTHistory. The clinical patient data used to evaluate the processing performance cannot be shared publicly due to institutional privacy restrictions regarding Protected Health Information (PHI).
