# Supplementary material for: RTHistory: An automated tool for the extraction and presentation of radiotherapy course history for continuity of care applications
Source: J Appl Clin Med Phys. 2026 Nov 14;27(11):e70910. doi: 10.1002/acm2.70910 (PMC13769382; doi:10.1002/acm2.70910)

External Beam Radiotherapy Treatment History

Patient Name: Doe^John

Date of Birth: January 01, 1900

Year	Treatment Start Date	Plan Label	Rx Dose	Dose per Fraction	Fractions Completed
2020	XXXX, 2020	i1RNeck	40.0 Gy	8.0	2/5
	XXXX, 2020	i2AntTongue	40.0 Gy	8.0	2/5
	XXXX, 2020	i3LNeck	40.0 Gy	8.0	2/5
	XXXX, 2020	A1RNeck	24.0 Gy	8.0	2/3
	XXXX, 2020	A2RNeck_1Fx	8.0 Gy	8.0	1/1

Plan Label:	i1RNeck
Prescribed Dose:	40.0 Gy
Dose Per Fraction:	8.0
Prescribed Fractions:	5
Treatment Start:	XXXX, 2020
Treatment End:	XXXX, 2020
Fractions Delivered:	2 / 5

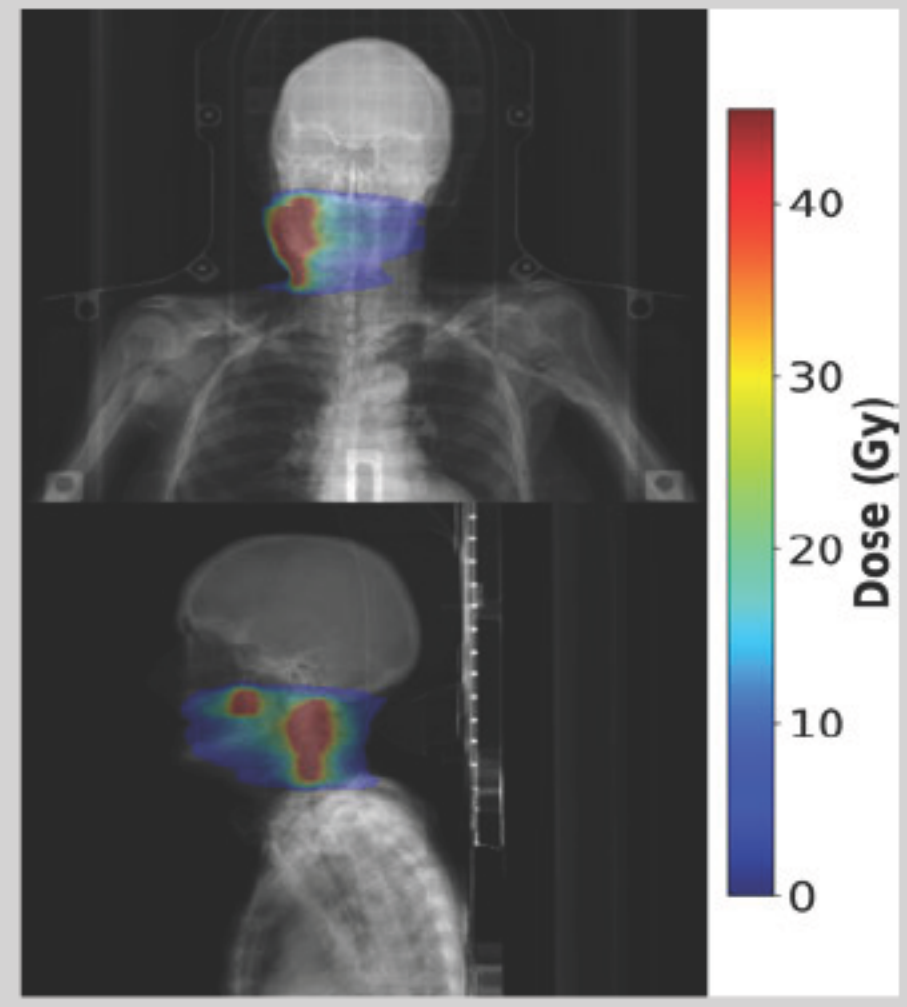

Plan Label:	i2AntTongue
Prescribed Dose:	40.0 Gy
Dose Per Fraction:	8.0
Prescribed Fractions:	5
Treatment Start:	XXXX, 2020
Treatment End:	XXXX, 2020
Fractions Delivered:	2 / 5

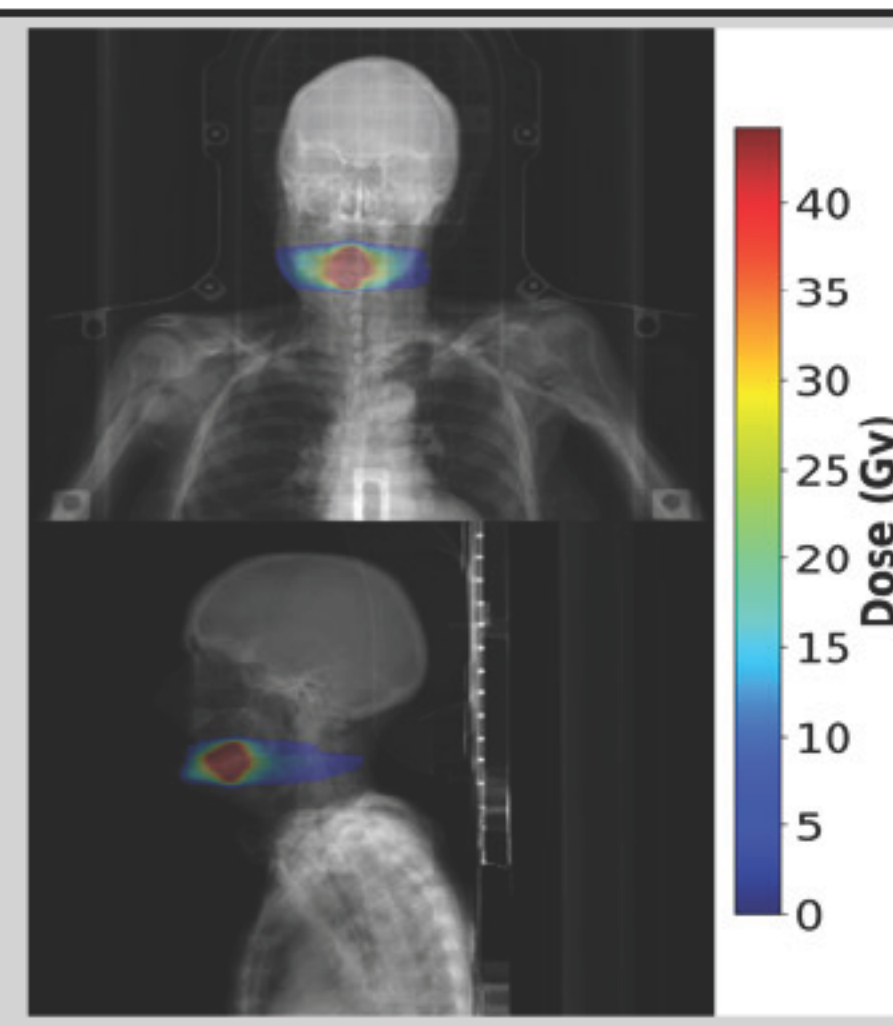

Plan Label:	i3LNeck
Prescribed Dose:	40.0 Gy
Dose Per Fraction:	8.0
Prescribed Fractions:	5
Treatment Start:	XXXX, 2020
Treatment End:	XXXX, 2020
Fractions Delivered:	2 / 5

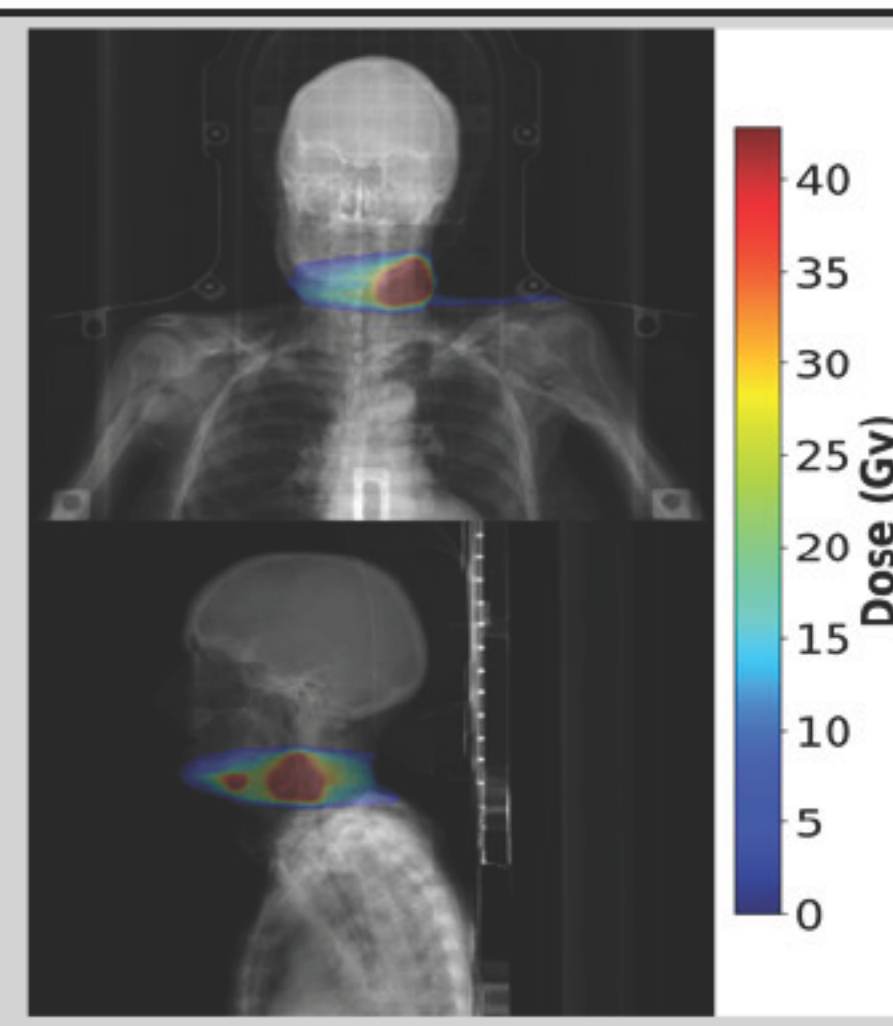

Plan Label:	A1RNeck
Prescribed Dose:	24.0 Gy
Dose Per Fraction:	8.0
Prescribed Fractions:	3
Treatment Start:	XXXX, 2020
Treatment End:	XXXX, 2020
Fractions Delivered:	2 / 3

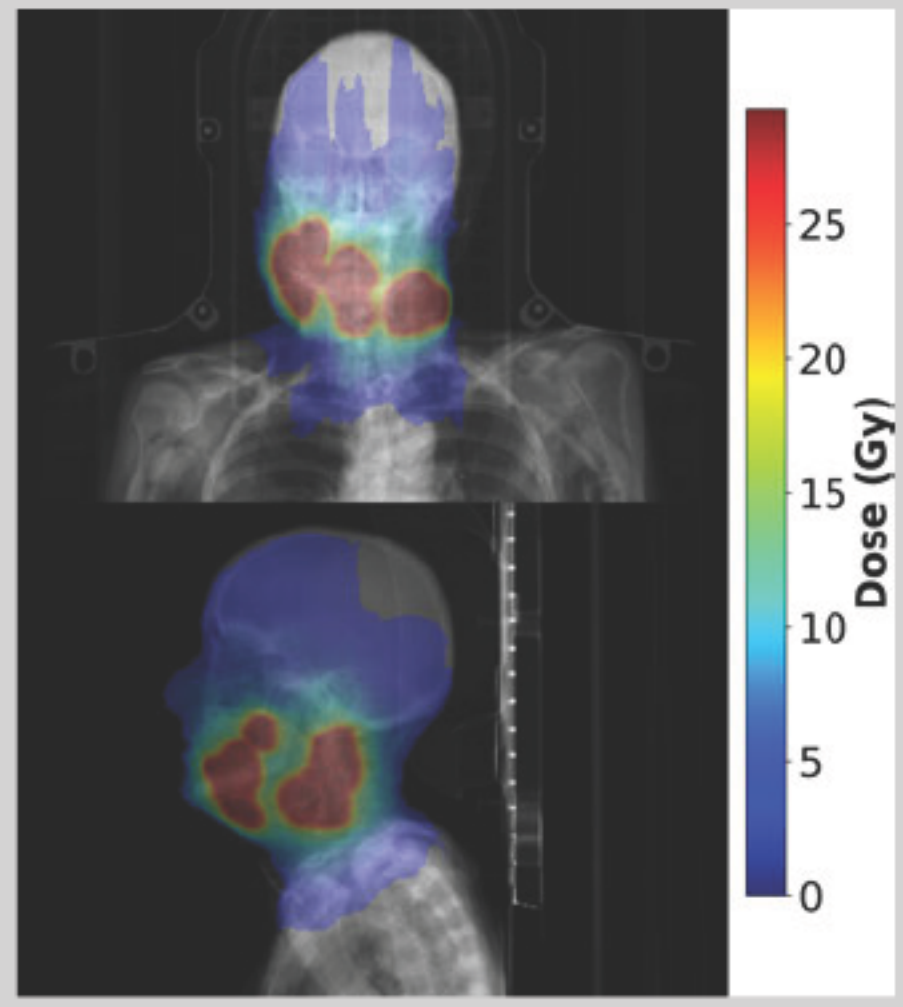

Plan Label:	A2RNeck_1Fx
Prescribed Dose:	8.0 Gy
Dose Per Fraction:	8.0
Prescribed Fractions:	1
Treatment Start:	XXXX, 2020
Treatment End:	XXXX, 2020
Fractions Delivered:	1 / 1

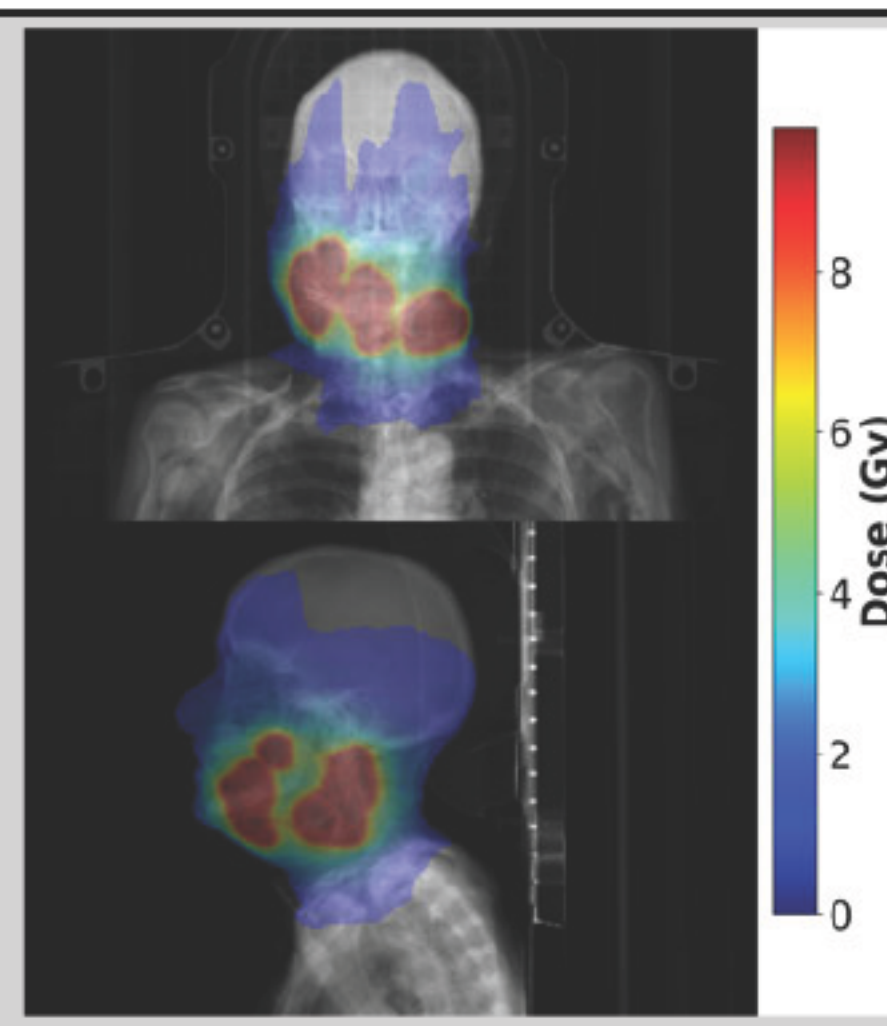

Supplement: Supplementary file 1 [file ACM2-27-e70910-s001.pdf]
